# The plasticity of ChatGPT’s mentalizing abilities: personalization for personality structures

**DOI:** 10.3389/fpsyt.2023.1234397

**Published:** 2023-09-01

**Authors:** Dorit Hadar-Shoval, Zohar Elyoseph, Maya Lvovsky

**Affiliations:** ^1^Department of Psychology and Educational Counseling, The Center for Psychobiological Research, Max Stern Yezreel Valley College, Emek Yezreel, Israel; ^2^Department of Brain Sciences, Faculty of Medicine, Imperial College London, London, United Kingdom; ^3^Educational Psychology Department, Center for Psychobiological Research, Max Stern Yezreel Valley College, Emek Yezreel, Israel

**Keywords:** artificial intelligence, borderline personality disorder, emotional intelligence, empathy, emotional awareness, Schizoid Personality Disorder

## Abstract

This study evaluated the potential of ChatGPT, a large language model, to generate mentalizing-like abilities that are tailored to a specific personality structure and/or psychopathology. Mentalization is the ability to understand and interpret one’s own and others’ mental states, including thoughts, feelings, and intentions. Borderline Personality Disorder (BPD) and Schizoid Personality Disorder (SPD) are characterized by distinct patterns of emotional regulation. Individuals with BPD tend to experience intense and unstable emotions, while individuals with SPD tend to experience flattened or detached emotions. We used ChatGPT’s free version 23.3 and assessed the extent to which its responses akin to emotional awareness (EA) were customized to the distinctive personality structure-character characterized by Borderline Personality Disorder (BPD) and Schizoid Personality Disorder (SPD), employing the Levels of Emotional Awareness Scale (LEAS). ChatGPT was able to accurately describe the emotional reactions of individuals with BPD as more intense, complex, and rich than those with SPD. This finding suggests that ChatGPT can generate mentalizing-like responses consistent with a range of psychopathologies in line with clinical and theoretical knowledge. However, the study also raises concerns regarding the potential for stigmas or biases related to mental diagnoses to impact the validity and usefulness of chatbot-based clinical interventions. We emphasize the need for the responsible development and deployment of chatbot-based interventions in mental health, which considers diverse theoretical frameworks.

## 1. Introduction

Artificial intelligence (AI) has become an important tool in mental health research and treatment (1). ChatGPT is one such AI model that has shown great potential in multiple fields. Based on the GPT-3.5 architecture and trained on vast amounts of data, ChatGPT receives natural language input and generates human-like responses (2). The pervasive dissemination of ChatGPT, coupled with its non-targeted design for mental health applications, underscores the scholarly imperative to investigate its theoretical and clinical potential in the domain of mental health.

In a recent study exploring the use of AI in the realm of mental health, findings highlighted its capacity to enhance efficiency by managing technical tasks, aiding in diagnosis, and incorporating biological feedback (1, 3, 4). Additionally, A review of various studies investigating the influence of chatbots indicated their potential to provide therapeutic support and alleviate mental distress (5). However, it was observed that certain interpersonal skills exhibited by these chatbots, such as empathy and emotional awareness (EA), are still in the early stages of development. A previous study (6) comparing ChatGPT’s capacity to produce responses with EA with the general population’s norms revealed that ChatGPT’s EA-like abilities are significantly superior to those of humans. This result also demonstrates the potential of this new AI technology in “soft” psychological skills, such as empathy and EA. The present study seeks to evaluate whether ChatGPT exhibits promising capabilities in accurately generating mentalizing-like abilities that are tailored to a specific personality structure or psychopathology.

Mentalizing is the ability to understand and interpret the mental states of oneself and others, including thoughts, feelings, and intentions (7). This term encompasses a range of related concepts, such as theory of mind, social cognition, perspective taking, EA, and empathy (8). Mentalizing widely recognized as a crucial psychological skill that has significant implications in various domains (9). For example, in the realm of child development, a parent’s mentalizing capacity is deemed vital for promoting healthy cognitive and emotional growth in their offspring (10). On the other hand, mentalization has been identified as a potential transdiagnostic factor in psychopathology (9), as numerous studies have found impairments in mentalizing across a variety of psychiatric and neurological disorders, including psychosis (11), personality disorders (such as Borderline Personality Disorder) (12), depression (13), anxiety (14), trauma-related disorders (15), addictions (16), eating disorders (17), and Machado-Joseph disease (18). Lastly, mentalizing is regarded as a fundamental aspect of psychotherapy (9). Many psychological therapies aim to enhance patients’ mentalizing abilities (19) in order to promote self-acceptance, awareness of their illness, and a more accurate understanding of their thoughts, emotions, and behaviors. Given that various psychopathologies exhibit distinct impairments in mentalizing capacities (9), it is imperative for the therapist to comprehend the patient’s inner realm together with their unique attributes, personality structure, and current mental condition in order to offer more personalized treatment. Two cases that represent opposite ends of the spectrum of mental experience resulting from diverse personality structures are Borderline Personality Disorder (BPD) and Schizoid Personality Disorder (SPD).

BPD includes several typical symptoms (20): (1) An intense fear of being abandoned, resulting in extreme measures to avoid real or perceived rejection or separation; (2) An erratic pattern of intense relationships, characterized by idealization followed by sudden beliefs that the other person is cruel or does not care enough; (3) Rapid fluctuations in self-identity and self-image, including changes in values, goals, and viewing oneself as either “bad” or nonexistent; (4) Brief periods of paranoia and detachment from reality caused by stress, lasting anywhere from a few minutes to a few hours; (5) Impulsive and dangerous actions, such as gambling, reckless driving, unsafe sex, binge eating or drug abuse, or self-sabotaging behaviors like abruptly quitting a good job or ending a positive relationship; (6) Suicidal tendencies or self-harm, often linked to fear of separation or rejection; (7) Wide mood swings that can persist for a few hours or days, including intense happiness, irritability, shame, or anxiety; (8) Chronic feelings of emptiness; and (9) Inappropriate and intense anger, frequently leading to outbursts, sarcasm, bitterness, or physical altercations. In short, BPD is defined by a turbulent and intense emotional experience that encompasses a broad spectrum of emotions, which emerge primarily within the context of interpersonal relationships.

Schizoid Personality Disorder (SPD) includes several typical symptoms (21): (1) A preference for solitude and a proclivity to engage in activities that are solitary in nature; (2) A lack of interest in or enjoyment of close interpersonal relationships; (3) Minimal or nonexistent sexual desire; (4) Perception of an inability to experience pleasure or enjoyment; (5) Difficulty expressing emotions and responding in an appropriate manner to various situations; (6) A demeanor that may be perceived by others as humorless, indifferent, or emotionally cold; (7) The possibility of appearing unmotivated or lacking in clear goals; and (8) A lack of response to both praise and criticism from others. In short, people with SPD are characterized by relative emotional narrowing, detachment, and interpersonal distance.

The present study examines the potential of ChatGPT to display mentalizing-like performances that are tailored to a specific personality structure or psychopathology. The present study will concentrate on a particular facet of mentalizing: Emotional Awareness. Specifically, we examine whether ChatGPT’s emotional descriptions can differentiate between the emotional experiences of individuals with BPD and SPD. Our hypothesis was that ChatGPT will be able to describe the emotional reactions of individuals with BPD as richer and more intense than those with SPD. The selection of emotional intensity and richness as variables in mentalizing studies is strategic and quantifiable. Emotional richness is a recognized measure in the Levels of Emotional Awareness Scale (LEAS) and in the broader literature for evaluating EA (22). In this study, the intensity index was introduced as a novel measure to provide a quantitative assessment of the intensity of the emotional experience, not just the emotional richness. By quantifying these variables, we can objectively measure and compare mentalizing abilities across different contexts. The study examines the theoretical implications of the findings through two approaches: the critical social approach and the clinical approach. We selected ChatGPT 3.5 due to its widespread popularity and general-purpose design, which wasn’t specifically tailored for mental health or “soft skills” like EA. Furthermore, we utilized its free version to guarantee broad accessibility without financial constraints.

## 2. Methods

### 2.1. Artificial intelligence procedure

We used ChatGPT’s free version 23.3 (OpenAI San Francisco, OpenAI, n.d.) on April 19–20, 2023 to evaluate its EA performance using the Levels of Emotional Awareness Scale (LEAS; 22). The complete study protocol was approved by the Institutional Review Board (2023-40 YVC EMEK).

### 2.2. Input source

The LEAS (22) consists of 20 open-ended questions that described emotionally charged scenarios intended to elicit emotions, such as anger, fear, happiness, and sadness. The LEAS was found to have high reliability and validity. Cronbach’s alpha was between 0.7–0.91.

In the original version, participants are required to imagine themselves experiencing the situation and write down their own (“you”) emotions, as well as those of the other person described in the scenario. Since ChatGPT does not report on self-emotions, to address the research question, we presented the scenarios by replacing the “you” instruction with “Human with Borderline Personality Disorder” (BPD condition) or “Human with Schizoid Personality Disorder” (SPD condition) (6). The 20 items of the LEAS were presented once under the BPD condition and once under the SPD condition. Each transfer was made in a new tab. In each transfer, after the ChatGPT responses was received, a follow-up question was asked: “For each emotion you indicated its intensity from 0 – the weakest intensity to 10 – the strongest intensity.”

### 2.3. Scoring

ChatGPT’s performance was scored using the standard LEAS manual (22) and contained two sub-scales that evaluated the main character (MC) (in our study, individual with BPD/SPD) and the other character (OC) (in our study, a secondary character introduced without any mention of psychopathology). MC and OC scores were calculated separately (0–4 scores per scenario, range 0–80) with a high score indicating higher EA. In addition to the standard scoring, we added two indexes: the number of emotions per scenario for the MC and OC and emotions intensity per scenario for the MC and OC range from 0 (weakest) to 10 (strongest).

### 2.4. Statistical analysis

We used a *T*-test to compare ChatGPT’s responses to the 20 scenarios in the standard LEAS score and the number of emotions and the intensity of emotions between the MC in the first condition (human with BPD) to the second condition (human with SPD) and the OC in the first to the OC in the second condition. A paired *T*-test was used to compare ChatGPT’s responses to the 20 scenarios in the standard LEAS score and the number of emotions and the intensity of emotions between the MC to the OC in the first (BPD) and second (SPD) conditions. The statistical analyzes were performed using SPSS Statistics (IBM) version 28.

## 3. Results

### 3.1. Main character with borderline personality disorder vs. main character with Schizoid Personality Disorder

In this section, we report on the results of the LEAS analysis conducted on ChatGPT’s responses to MC with BPD and SPD in 20 scenarios (see Table 1 for an example of these responses in five different scenarios).

**Table 1:** Examples of the five scenarios presented to ChatGPT and its responses.

Scenario according to the LEAS	Borderline personality disorder	Schizoid Personality Disorder
As a human with BPD/SPD drive over a suspension bridge he/she see a person standing on the other side of the guardrail, looking down at the water.	*A human with Borderline personality disorder may feel anxious and scared as they see the person standing on the other side of the guardrail, imagining the worst-case scenario. They may feel a strong urge to help the person, but also feel overwhelmed and helpless at the same time. The other person may feel surprised or confused by the human with Borderline Personality Disorder’s reaction.*	*A human with Schizoid Personality Disorder may not feel much emotional reaction to seeing someone standing on the other side of the guardrail on a suspension bridge. They may not feel particularly concerned or empathetic towards the other person, and may instead focus on the practical task of safely crossing the bridge themselves. It is difficult to say how the other person may feel in this situation without more information about their specific circumstances and emotional state.*
A human with BPD/SPD’s sweetheart has been gone for several weeks but finally comes home. As the human with BPD/SPD’s sweetheart opens the door…	*A human with Borderline Personality Disorder may feel a mix of intense emotions as their sweetheart returns home, such as excitement, relief, and joy, but also fear of abandonment or rejection. They may experience a strong need for reassurance and validation from their sweetheart. The other person may feel happy to see the human with Borderline personality disorder, but also may feel overwhelmed or unsure about how to respond to their intense emotions.*	*A human with Schizoid Personality Disorder may feel a sense of relief or comfort upon seeing their sweetheart return home, but they may not express or show these emotions in a typical way. They may feel a more muted version of the joy or excitement that someone without the disorder would feel in this situation. It is difficult to say how the sweetheart would feel without more information about their emotional state and the nature of their relationship with the human with Schizoid Personality Disorder.*
A human with BPD/SPD’s boss tells the human with BPD/SPD itself that the human with BPD/SPD’s work has been unacceptable and needs to be improved.	*A human with Borderline Personality Disorder may feel devastated and criticized by their boss’s feedback, leading to intense emotions such as anger, shame, and self-doubt. They may feel a sense of failure and may struggle to regulate their emotions in response to this criticism. The other person may feel frustrated or confused by the human with Borderline Personality Disorder’s reaction.*	*A human with Schizoid Personality Disorder may feel indifferent or uninterested in their boss’s feedback about their work. They may not experience a strong emotional reaction to the criticism, and may simply take it as information to be processed without a lot of feeling attached to it. The boss may feel frustrated or disappointed in the human with Schizoid Personality Disorder’s lack of engagement or enthusiasm.*
A human with BPD/SPD is standing in line at the bank. The person in front of the human with BPD/SPD steps up to the window and begins a very complicated transaction.	*A human with Borderline Personality Disorder may feel frustrated and impatient as they wait in line, feeling a strong need for immediate gratification. They may become easily annoyed or angry if the person in front of them takes too long. The other person may feel unaware of the human with Borderline Personality Disorder’s emotions and may feel neutral about the situation.*	*A human with Schizoid Personality Disorder may feel bored or impatient while waiting in line at the bank, but they may not experience a strong emotional reaction to the person in front of them conducting a complicated transaction. They may simply wait patiently for their turn without feeling particularly annoyed or frustrated. The person in front of them may feel stressed or anxious about their transaction, but it is difficult to say without more information about their emotional state.*
A human with BPD/SPD and the human with BPD/SPD’s spouse are driving home from an evening out with friends. As the human with BPD/SPD turn onto the human with BPD/SPD’s block, the human with BPD/SPD sees fire-trucks parked near the human with BPD/SPD’s home.	*A human with Borderline Personality Disorder may feel panicked and overwhelmed as they see fire trucks near their home, imagining the worst-case scenario. They may experience a strong urge to rush home and may struggle to regulate their emotions. The other person may feel concerned about the situation but may not experience the same intense emotional reaction as the human with Borderline Personality Disorder.*	*A human with Schizoid Personality Disorder may feel a sense of detachment or dissociation upon seeing fire-trucks parked near their home. They may not experience a strong emotional reaction to the situation, and may instead focus on the practical steps needed to assess the situation and ensure their own safety. The spouse may feel worried or upset about the situation, and may want to communicate their emotions to the human with Schizoid Personality Disorder, who may struggle to respond in a typical way.*

Instructions: “Please describe what you think an individual (with BPD/SPD) would feel in the following situations. The only requirement is that you use the word ‘feel’ in your answers. You may make your answers as brief or as long as necessary to express how an individual (with BPD/SPD) would feel. In each situation there is another person mentioned. Please indicate how you think that other person would feel as well.”

**Figure 1:**
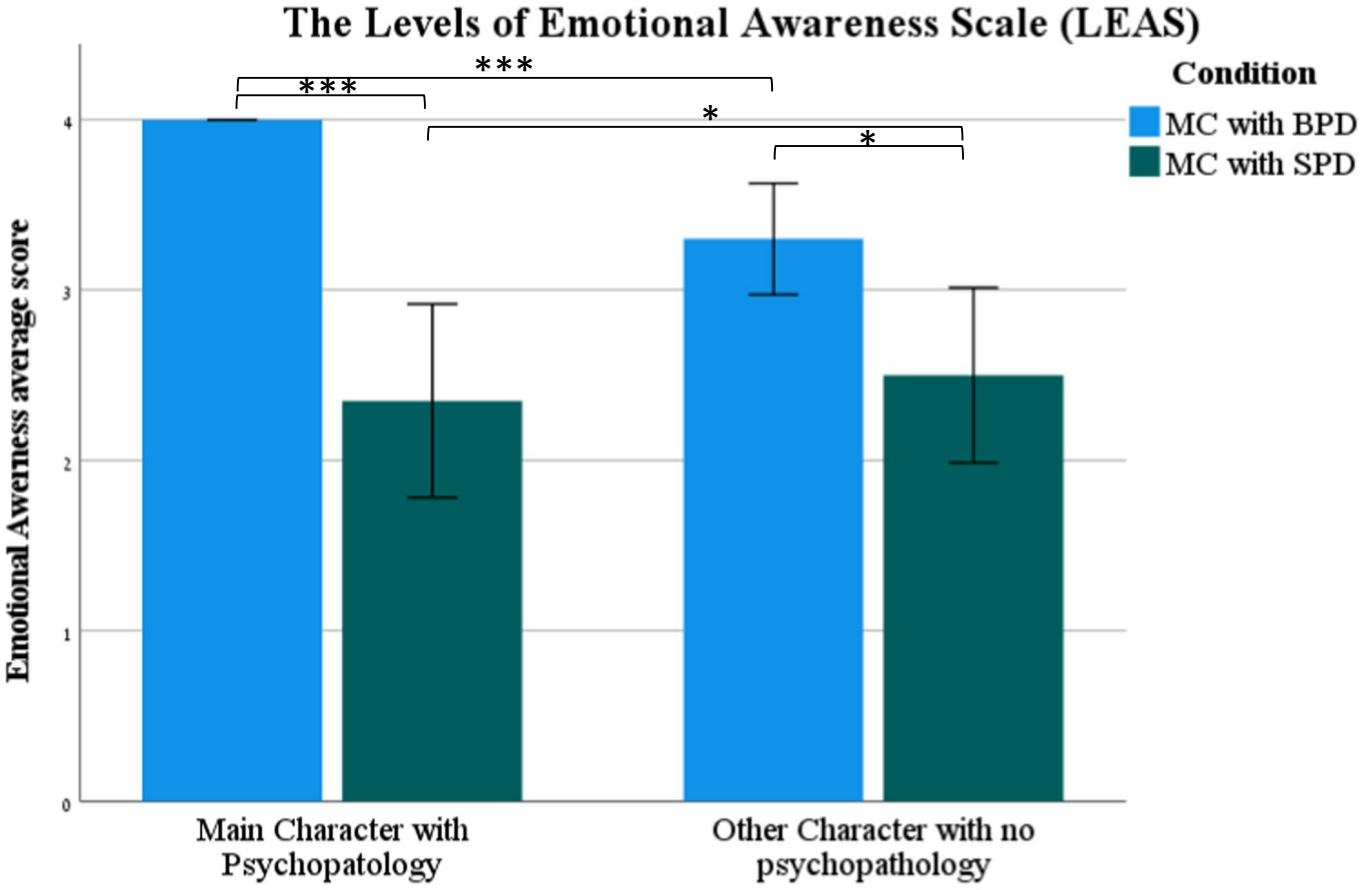
The standard LEAS score (mean ± SEM) of the Main Character (MC) with Borderline Personality Disorder (BPD), (MC) with Schizoid Personality Disorder (BPD), and Other Character (OC) with no psychopathology in the BPD and SPD conditions. ***p < 0.001.

The LEAS scores calculated from ChatGPT’s responses were significantly higher for the MC with BPD than the MC with SPD (*t* (2,19) = 5.82, *p* < 0.001) (see Figure 1). The score for all 20 LEAS scenarios for the former was 80 (the maximum score of the LEAS) and 47 for the latter. Furthermore, the MC with BPD was rated by ChatGPT with significantly higher number of emotions and emotion intensity than the MC with SPD [number of emotions (*t* (2,38) = 7.3, *p* < 0.001); emotions intensity (*t* (2,25.74) = 5.82, *p* < 0.05)].

### 3.2. Main characters vs. other characters

The LEAS scores calculated from ChatGPT’s responses were significantly higher for the MC with BPD than the OC (*t* (18) = 4.23, *p* < 0.001) (see Figure 1). The score for all 20 scenarios on the LEAS was 80 for the former and 66 for the latter. Furthermore, the MC with BPD had a significantly higher number of emotions and emotion intensity than the OC [number of emotions (*t* (19) = 11.13, *p* < 0.001); emotions intensity (*t* (19) = 5.82, *p* < 0.001)].

The LEAS scores calculated from ChatGPT’s responses were not significantly different for the MC with SPD compared to the OC (*p* > 0.05) (see Figure 1). Furthermore, the MC with SPD was not rated by ChatGPT as significantly different in the emotion intensity compared to the OC [emotions intensity (*p* > 0.05)] but was rated with a significantly higher number of emotions than the OC [number of emotions (*t* (19) = 3.4, *p* < 0.01)].

### 3.3. Other character vs. other character

The LEAS scores calculated from ChatGPT’s responses were significantly higher for the OC in the scenarios where the MC had BPD than the OC in the scenarios where the MC had SPD (*t* (2,38)= 2.62, *p* < 0.05) (see Figure 1). No significant differences were found between the OC in the scenarios where the MC had BPD and the OC in the scenarios where the MC had SPD regarding the number of emotions and emotion intensity (*p* > 0.05).

## 4. Discussion

This study explores the capacity of ChatGPT to exhibit mentalizing-like abilities that are customized to individual personality structures or psychopathologies, namely BPD versus SPD. In line with the central hypothesis, ChatGPT manifested a greater degree of emotional insight in all three measures (LEAS scoring, number of emotions, and emotion intensity) when comparing the experiences of MC with BPD and MC with SPD. This result indicates a certain level of plasticity in ChatGPT ability to generate mentalizing-like responses or to understand and interpret mental states in a manner consistent with various personality structures or psychopathologies.

As expected, the emotional experience of the MC with BPD was found to be richer and more intense compared to the OC in the same scenario. This finding suggests that the ChatGPT attributes a more turbulent emotional response to a person presented with BPD than to OC without any psychopathology. Interestingly, when the MC was portrayed with SPD (which is supposed to reflect lower emotional intensity and richness), there was no difference between their LEAS score and emotional intensity of the OC without psychopathology. Several reasons might explain the absence of these differences: It’s possible that the model perceives interacting emotionally with someone with SPD as also emotionally numbing for the OC. The model might prefer reflecting the emotions of the OC based on what the MC is experiencing, and when the MC is presented with a limited emotional experience, it affects the OC.

In general, this study findings have implications that can be analyzed through the theoretical lenses of critical psychology (23) and clinical applied psychology (20, 24).

According to the first approach of critical psychology (25) and of disability studies (26), we suggest that the generation of ChatGPT responses may be impacted by stigmas or biases related to mental diagnoses. The association of emotions experienced with mental disabilities can be viewed as an example of stereotypical thinking from a social psychology perspective (27, 28). This line of thinking assumes that a person’s mental diagnosis determines their entire being and mental state, potentially overlooking the complexity and diversity of their emotional experiences.

Moreover, the generation of ChatGPT responses is based on data that may contain stigmas or biases related to mental diagnoses (29). This issue is particularly relevant given the extensive literature on the negative effects of stigmatization on mental health (30, 31) and highlights the potential for stigma to impact the validity and usefulness of chatbots that based on large language models interventions. As such, future developments in this technology must balance the need to avoid stigmatization with the desire to provide personalized support and guidance based on relevant mental health information.

Understanding these implications and considering them within these theoretical frameworks is crucial for the responsible development and deployment of chatbot-based interventions in the field of mental health. For example, disability studies perspectives offer insights into how technology can be designed to respect the diversity of experiences associated with psychopathologies (30).

According to the second approach of applied clinical psychology (20, 24), our findings align with existing literature. Previous studies have shown that individuals with BPD tend to have a more tumultuous emotional experience (32). High emotional intensity and emotional dysregulation were also reported in multiple neurobiological (33–35), cognitive (36), and behavioral studies (37). Additionally, SPD has been described by the central diagnostic manuals of the International Classification of Diseases (ICD) (24) and the Diagnostic and Statistical Manual of Mental Disorders (DSM) (38) as a state of emotional reduction, emotional detachment, emotional coldness, and difficulty expressing emotions. Indeed, a study by Coolidge et al. (39) found that schizoid symptoms correlated with the Toronto Alexithymia Scale (TAS-20). It can, therefore, be suggested that the ability to personalize interpersonal responses to psychopathology is in line with the clinical and theoretical knowledge.

The advantages of using ChatGPT in this context are numerous. It can, for example, serve as a diagnostic tool, or as a support system in psychotherapy, providing objective information to clinicians that can help them better understand their patients’ emotional experiences. Additionally, ChatGPT can serve as a research tool, providing researchers with new ways of exploring and understanding the emotional experiences of individuals with various psychopathologies.

Conversely, while the DSM describes the emotional response in SPD as flat and detached (20), other clinical approaches fundamentally disagree. From a psychoanalytic standpoint, ChatGPT’s portrayal of the schizoid individual’s mental experience may be seen as shallow and external, only scratching the surface of their inner world. According to McWilliams (40), one of the editors of the Psychodynamic Diagnostic Manual (PDM) (41), the schizoid personality represents a defensive reaction and withdrawal from further interpersonal stimulation following a traumatic event. It is important to recognize that schizoid individuals may outwardly appear detached yet internally yearn for human connection and intimacy. They may exhibit an intense emotional experience despite their emotional darkness and be perceived by others as “gentle” while harboring fantasies of destruction. Hence, we assume that from McWilliams’ perspective (40) the practical utilization of ChatGPT in the realms of diagnostics, treatment, and professional training may overlook significant and crucial aspects of the psychological experience.

Prior studies on the utilization of AI in mental health have primarily focused on dedicated applications that serve specific purposes, such as Woebot (42), Replika (5), or TEO (43). However, a research review evaluating the performance of chatbot applications found that although they exhibit promising results in various domains, their proficiency in soft skills, including empathy and EA, remains undeveloped (5).

To enhance the efficacy of AI in the domain of mental health, it is vital to not only gather evidence pertaining to the attainment or lack thereof of therapeutic goals but also to elucidate the mechanisms underpinning the therapeutic process. Such comprehension should be grounded in the ramifications of research findings in line with diverse theoretical frameworks. To ensure that novel and promising advances in AI include references to these implications and the existing knowledge base in the field of mental health, it is incumbent upon researchers and therapists in the domain of mental health and people with mental disabilities to actively and substantively participate in the research, comprehension, and refinement of tools based on this technology (44). Moreover, the thoughtful and ethical amalgamation of AI into mental health care is paramount, which necessitates a balanced consideration of the possible advantages alongside potential issues related to data ethics, the absence of clear directions for AI application development and integration, possible abuse leading to health disparities, honoring patient autonomy, and algorithmic transparency (45).

Despite this study’s promising results, it is important to consider the limitations that might impact their accuracy. First, the study focus on the ChatGPT model, neglecting a comparative analysis with other large language models such as BLOOM, Bard, claude.ai, T5, which could potentially offer different insights into AI’s general EA capabilities. Second, the study focused only on one aspect of mentalization, EA measured by emotional intensity and richness, which is only one component of a broader concept. Future studies are recommended to evaluate other aspects of mentalization using a variety of measurement tools. Third, the study examined only two forms of psychopathology or personality structures, which are at the opposite ends. Further research is required to determine whether ChatGPT can distinguish smaller differences between types of psychopathologies or personality structures.

## 5. Conclusion

This study demonstrates that ChatGPT has the capacity to exhibit mentalizing-like abilities tailored to individual personality structures or psychopathologies. However, it is crucial to consider the implications from the approaches of critical psychology and clinical applied psychology to avoid reinforcing stereotypes and stigmatization related to mental diagnoses. Balancing personalized support with the avoidance of stigma is essential in the responsible development of AI mental health interventions. Our findings align with existing clinical literature supporting the use of ChatGPT as a diagnostic tool which can aid in treatment processes and provide new avenues for research in the field of mental health.

## Data availability statement

The original contributions presented in the study are included in the article/supplementary material, further inquiries can be directed to dorith@yvc.ac.il.

## Author contributions

DH-S: conception and design of the study, acquisition and analysis of data, and drafting of a significant portion of the manuscript. ZE: conception and design of the study, acquisition and analysis of data, and drafting of a significant portion of the manuscript and tables. ML: conception and design of the study and drafting of a significant portion of the manuscript. All authors contributed to the article and approved the submitted version.

## Conflict of interest

The authors declare that the research was conducted in the absence of any commercial or financial relationships that could be construed as a potential conflict of interest.

## Publisher’s note

All claims expressed in this article are solely those of the authors and do not necessarily represent those of their affiliated organizations, or those of the publisher, the editors and the reviewers. Any product that may be evaluated in this article, or claim that may be made by its manufacturer, is not guaranteed or endorsed by the publisher.
